# Chest pain management in acute coronary syndrome and chronic stable angina with diluted xylocaine injections

**DOI:** 10.6026/973206300212977

**Published:** 2025-09-30

**Authors:** Sarvesh Jain, Roopali Jain, Arvind Diwakar Jain

**Affiliations:** 1 Department of Anaesthesiology and Critical Care and Pain Medicine, Government Bundelkhand Medical College, Sagar, Madhya Pradesh, India; 2 Department of (Diploma in Gynecology and Obstetrics), Gynaecologist, Pain Clinic, Sagar, Madhya Pradesh, India; 3 Private Practitioner, Lalitpur, Uttar Pradesh, India

**Keywords:** Pain portal block, heart attack, myocardial infarction, chest pain

## Abstract

Pain following myocardial ischemia is a significant clinical concern, contributing to increased sympathetic tone, mortality and morbidity. Standard management includes beta blockers, nitrates, aspirin-clopidogrel and opioids. We propose a robust pain portal block technique using 0.5% lignocaine at designated sites on the chest wall and upper limb. This method offers rapid and effective analgesia for both cardiac and non-cardiac chest pain, extending beyond lignocaine's typical duration of action. Early implementation of this technique may positively impact the management and outcomes of acute myocardial infarction.

## Views:

Currently, we are facing an epidemic of myocardial ischemia and infarction, where diminished blood flow to the myocardium may lead to an emergency known as acute coronary syndrome (ACS) and chronic chest pain on exertion known as stable coronary artery disease (CAD). Pain leads to increased sympathetic tone, which eventually leads to tachycardia, hypertension, more myocardial oxygen requirement, increased anxiety and more patient suffering [1]. Apart from the need for pain relief on humanitarian grounds, it may increase the death rate in an acute situation [2]. The current standard treatment for this is beta blockers, nitrates, aspirin clopidogrel combination and opioid analgesic. Hence, tachycardia and hypertension, increased oxygen demand and pain all can be reduced [1, 2]. We recommend a novel approach to chest pain relief of cardiac and noncardiac origin. Pre designated areas termed as T1, T2 and alpha 1 are part of nomenclature used for DSCB (distal sodium channel block) for treatment of different musculoskeletal pain condition, where we need to inject in 1 mL of 0.5% lignocaine over the midsternal point, 1 mL of 0.5% lignocaine over 6th rib mid axillary line on left side and 2 mL of 0.5% lignocaine at first web space i.e. the belly of adductor pollicis muscle. We observed good to excellent pain relief for acute coronary syndrome and chest pain of both cardiac and non-cardiac origin within 20 minutes. First and second point injections can also provide relief for rib fractures, flail chest, pneumonia and other chest wall disorders. Injecting lignocaine for nerve block and various painful conditions is common practice at our institution [1, 2]. Therefore, it is of interest to report chest pain management in acute coronary syndrome and chronic stable angina with diluted xylocaine injections. Neural therapy means the injection of local anesthetics for therapeutic intent. The injection can be made at the scar, skin, ganglia and nerve [3, 4]. Out of so many proposed mechanisms of action of neural therapy; one is that infiltrating terminal branches of the spinal nerve at a fixed site, leads to decreased sympathetic afferent stimulus to the spinal cord. This will lead to a decrease in the sympathetic efferent outflow. This infiltration has to be done twice a week for at least the 6th week. But the effect may last for days to weeks even after single infiltration. Its proponent claims that it has potential to treat diabetes, kidney failure, cancer and other systemic disorder too [4]. Being thoracic viscera, heart has both sympathetic and parasympathetic supply. Pain carrying sensation reaches up to the lower cervical and upper thoracic sympathetic ganglion. Cervical preganglionic fiber re-enters spinal cord at the thoracic spinal cord. The heart has nerve supply from T1 T4. Since the T1 dermatome also supplies the medial aspect of the forearm and arm, so sometimes anginal pain radiates to the upper limb too. The chest wall is innervated by the T1 T11 dermatome via the intercostal nerve [5].

We inject to abolish pain sensation reaching through sympathetic afferent fibers from the heart and chest wall. This is the predesignated location 1) Midsternal point, 2) 6th rib mid axillary line on the left side, 3) First web space i.e. belly of adductor pollicis muscle This abolition for time being, breaks the vicious cycle of increased pain sensation reaching along with sympathetic fibers to the spinal cord and the resultant increased tone of sympathetic efferent, in other words, we infiltrate terminal branches of the spinal nerve to produce analgesia [6]. The important thing to remember local anaesthetic solutions should be diluted to block only the autonomic fibers, not the motor and sensory fibers. A mostly single injection is sufficient to provide relief in emergencies. It was our observation that no changes in vitals, hemodynamics and Electrocardiogram (ECG) occur. There is a theoretical possibility of slight hypotension. A conclusive statement about its efficacy should be made only after 20 minutes. Lignocaine is an amide local anaesthetic that was invented in 1946. It has a very long and safe history for use as an infiltration, intrathecal and intravenous route. Its effects on autonomic fibers last for 2-3 hours [7, 8]. The weaker strength of lignocaine injection produces analgesia for days and weeks. Although this analgesia cannot be explained based on the pharmacokinetics and pharmacodynamics of lignocaine, authors have used this technique for low back pain with radiation (L5 radiculopathy), just in the form of an injection at the anterior and posterior to the lateral malleolus. For neck pain and frozen shoulder have been used successfully in a lot of patients. Authors also claim its efficacy in fractured rib, herpes zoster, flail chest and other chest wall muscular skeleton pain disorders [9]. Sometimes patients complain of light headaches, which respond very well to assurance. This intervention is known to produce a block at the pain portal, so it should be named a physiological pain portal block. Since local anesthetics show their action by blocking the sodium channel at the neuronal membrane and the injection site is always distal to dorsal root ganglion and cranial nerve nuclei, so it can be aptly named as distal sodium channel block (DSCB) [10]. For acute myocardial infarction pain, chronic cardiac pain conditions and acute and chronic somatic chest pain conditions, physiological pain portal block or DSCB is an easy, inexpensive, predictable and highly reproducible option of producing analgesia [11]. Due to its excellent safety margin, we recommend its use in every ACS and stable CAD. The magnitude of opioid related respiratory depression can be greatly reduced and the dose of the opioid required to treat such conditions, can also be reduced.
